# Iodine images in dual energy CT: A monocentric study benchmarking quantitative iodine concentration values of the healthy liver

**DOI:** 10.1371/journal.pone.0270805

**Published:** 2022-07-14

**Authors:** Stefanie Beck, Laurenz Jahn, Dominik Deniffel, Isabelle Riederer, Andreas Sauter, Marcus R. Makowski, Daniela Pfeiffer

**Affiliations:** 1 Department of Diagnostic and Interventional Radiology, Klinikum Rechts der Isar, Technische Universität München, Munich, Germany; 2 Department of Diagnostic and Interventional Neuroradiology, University Hospital RWTH Aachen, Aachen, Germany; 3 Department of Diagnostic and Interventional Neuroradiology, Klinikum Rechts der Isar, Technische Universität München, Munich, Germany; Northwestern University Feinberg School of Medicine, UNITED STATES

## Abstract

Dual energy computed tomography (DECT) allows the quantification of specific materials such as iodine contrast agent in human body tissue, potentially providing additional diagnostic data. Yet full diagnostic value can only be achieved if physiological normal values for iodine concentrations are known. We retrospectively evaluated abdominal DECT scans of 105 patients with healthy liver between March and August 2018 (age 17 to 86 years, 43 female and 62 male). The iodine concentrations within ROIs of the liver parenchyma as well as of the abdominal aorta and main portal vein were obtained. We evaluated the absolute iodine concentration and blood-normalized iodine concentrations relating the measured iodine concentration of the liver parenchyma to those of the supplying vessels. The influence of age and gender on the iodine uptake was assessed. The absolute iodine concentration was significantly different for the male and female cohort, but the difference was eliminated by the blood-normalized values. The average blood-normalized iodine concentrations were 2.107 mg/ml (+/- 0.322 mg/ml), 2.125 mg/ml (+/- 0.426 mg/ml) and 2.103 mg/ml (+/- 0.317 mg/ml) for the portal vein normalized, aorta normalized and mixed blood normalized iodine concentrations, respectively. A significant negative correlation between the patients’ age and the iodine concentration was detected only for the blood-normalized values. A physiological range for iodine concentration in portal venous phase contrast enhanced DECT images can be defined for absolute and blood-normalized values. Deviations of blood-normalized iodine concentration values might be a robust biomarker for diagnostic evaluation. Patient age but not the gender influences the blood-normalized iodine concentrations in healthy liver parenchyma.

## Introduction

Dual energy computed tomography (DECT) uses two separate energy sets to examine differing attenuation properties and has a significant advantage over traditional single energy CT systems [1, 2]. It allows, among many other clinical applications, the detection, separation and quantification of contrast agent such as iodine in solid organs and lesions [3–5] and therefore the generation of material density iodine images. Studies have demonstrated that DECT can quantify iodine with high accuracy, showing maximum deviations of 5–10 percent from the actual iodine concentrations [6–9]. The clinical launch of such dual energy CT systems, therefore, allows the assessment of iodine concentrations thorough the different organs and parenchyma in clinical routine CT imaging, offering the possibility to gain additional diagnostic information by analyzing the absorption of iodine in organs and lesions.

So far, clinical approaches have been studied in different fields, such as pulmonary diseases [10], renal masses [11], lymphadenopathy [12], thyroid nodules [13] and cardiology [14] between others. In the past, several studies have analyzed the relation between hepatic enhancement values and iodine concentration of the injected contrast agent in multiphase contrast enhanced CT, in order to assess the quantitative effects of contrast material concentration on hepatic parenchymal and vascular enhancement in terms of variations in the HU values [15, 16].

Through the direct quantification of iodine concentration in the liver parenchyma in terms of mg/ml, it is to be expected that the quantification of iodine concentration of the liver parenchyma can offer additional diagnostic benefit to CT examinations of the liver tissue.

Nevertheless, the additionally information of quantitative iodine concentration offered by material density iodine images does not provide full diagnostic value if physiological normal values for quantitative iodine concentrations have not been defined.

In order to obtain diagnostically relevant information, it is essential to establish the distribution of physiologically normal values of iodine concentration after contrast agent uptake in the liver parenchyma and to define their dependency on variables such as gender and age, since these relationships are necessary for interpretation of the iodine concentration in terms of pathology.

The purpose of this study was to establish the quantitative iodine concentration range of healthy liver parenchyma in portal venous phase Material Density (MD) iodine images in DECT and asses the effects of gender and age.

## Methods

### Study cohort

The retrospective study was approved and conducted in accordance with the guidelines of the Ethical Committee of the Klinikum rechts der Isar, Technische Universität München and informed consent was waived by the local institutional review board. We retrospectively searched our department’s computerized clinical database for all contrast enhanced portal venous phase abdominal DECT with material density iodine images from March 2018 to August 2018. Out of these images only those patients with a healthy liver were included in our study cohort. The diagnosis of healthy liver was defined by liver function blood analysis, considering only patients with normal GOT (glutamic oxaloacetic transaminase), GPT (glutamic pyruvate transaminase) and GGT (gamma glutamyl transferase), the most common liver blood test parameters in clinical routine. Furthermore, we included only those patients in our study cohort, that did not have any further known diseases that could possibly influence the liver metabolism, such as liver diseases (e.g., hepatitis, steatosis, cirrhosis, tumors, etc.) metabolic diseases (e.g. metabolic syndrome, diabetes, etc.) or cardiac diseases with reduced cardiac output (e.g. cardiac insufficiency, cardiac arrythmia, portal venous hypertonus, etc.).

And finally, the clinical patient history and lab results were corroborated and correlated with the CT images by 2 radiologists with 4 and 9 years of experience in interpreting abdominal CT on a PACS system. A total of 143 patients were included in the initial study cohort, among which 38 were then excluded because of strong artefacts or due to contrast agent administration not according to standard CT protocol used in the study. This yielded a final study population of 105 patients (62 male and 43 female) aged between 17 and 86 years and with a mean age of 55.56 years. Within the female group the mean age was 54.24 years and ages ranged between 17 and 86 years. Within the male group the mean age was 56.11 years and the ages ranged from 19 to 81 years.

### CT protocol

All CT examinations of the abdomen were acquired on a dual-layer dual-energy 64-channel CT scanner (IQon, Philips Healthcare, Cleveland, OH, USA) according to our institutional protocol. Contrast material was injected via a 20 G catheter into an ante-cubital vein with a rate of 2,5 ml/s using a dual syringe injection system (Stellant, MEDRAD, Indianola, Pennsylvania), followed by a 50 ml saline chaser. Portal venous phase images were obtained 70 s following intravenous administration of contrast material (80 ml Ultravist 370 MCT, Bayer Vital GmbH, Leverkusen, Germany). The scan was performed craniocaudally with a pitch of 0.9, tube voltage of 120 kVp, a 64x0.625-mm detector configuration, a rotation time of 0.33 seconds and an average CTDIvol of 7.8 mGy. All data sets were reconstructed in axial view with slice thickness of 5 mm and a 512-image matrix with an iDose image reconstruction algorithm level 2.

### CT image analysis

Image Analysis was performed using a commercially available spectral workstation (IntelliSpace Portal (v. 8.0.2), Philips Healthcare, USA) ensuring a high accuracy of iodine quantification [17]. The iodine concentrations are derived from photoelectric and Compton scatter attenuation base images, which are calculated from the high and low energy attenuation data using iterative reconstruction methods [18].

We used region-of-interest (ROI) measurements of iodine concentration on 5 mm thick portal-phase contrast enhanced material density iodine images. The iodine concentration of the liver parenchyma was measured by placing 3 ROIs of 1.5 cm^2^ in different points of the liver parenchyma. Two of the three ROIs were set in segments 4b and 7 of the right hepatic lobe and one ROI was set in segment 3 of the left hepatic lobe. All ROIs were placed with care to exclude macroscopic hepatic vessels.

The three measured iodine concentration values of a patient x are referred to as *I_ROI s.3_ (x)*, *I_ROI s.4b_ (x)* and *I_ROI s.7_ (x).*

The absolute iodine concentration of the liver parenchyma of a patient x, *I_L_abs_(x)*, was defined as the average value of the 3 measured iodine concentration values of the three ROIs *I_ROI s.3_ (x)*, *I_ROI s.4b_ (x)* and *I_ROI s.7_ (x)*.


$$I_{L\_abs}(x)=\frac{I_{ROI\;s.3}(x)+I_{ROI\;s.4b}(x)+I_{ROI\;s.7}(x)}{3}$$ (1)

**Fig 1:**
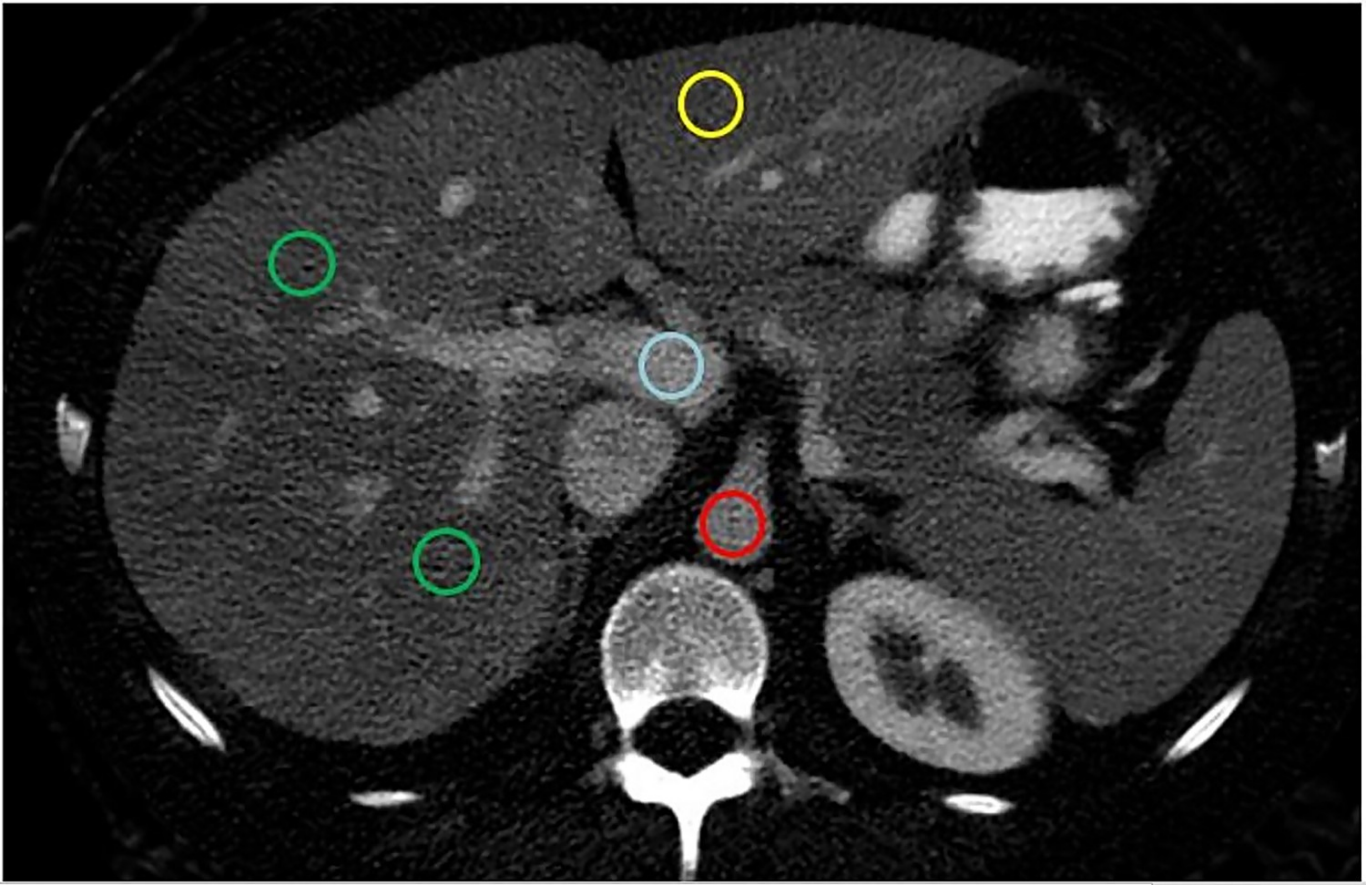
Dual energy CT iodine image, indicating the placement of the ROIs. The 5 mm thick slice of a DECT iodine image in portal venous phase image at the level of the coeliac axis shows five ROIs, all with an approximate area of 1.5 cm^2^. Two ROIs (green) are placed in the right hepatic lobe in segments 4b and 7, one ROI (yellow) in the left hepatic lobe in segment 3, one ROI (blue) in the portal vein, and one in the aorta (red).

The iodine concentration of the aorta *I_aorta_(x)* and the portal vein *I_portalvein_(x)* were measured by placing ROIs at the level of the coeliac axis. For the portal and aortal measurements, the largest possible circular ROI enclosed within the vessel lumen was used (Fig 1).

### Calculation of blood-normalized iodine concentrations

The iodine contrast agent is administered to the patients intravenously and carried to the liver through the circulatory system, thus through the aorta and the portal vein. Therefore, the iodine uptake of the liver parenchyma is highly influenced by the iodine concentration of the blood within these supplying vessels. Due to the individual and distinct properties of the circulatory system of different patients, the iodine concentration within the blood supplying vessels in portal venous phase may vary between patients, leading to a possible confounding bias.

Thus, we defined a blood-normalized iodine concentration of the liver *I_L_bn_(x)* that relates the measured absolute iodine concentration of the liver parenchyma *I_L_abs_(x)* to the measured absolute iodine concentration of the supplying blood vessels, aorta and portal vein, as these directly deliver the contrast agent supply of the liver.

The blood normalized iodine concentration of the liver *I_L_bn_(x)* is obtained by weighting the absolute iodine concentration of a patient’s liver *I_L_abs_(x)* with the iodine concentration of the same patient’s blood supplying vessels *I_blood_(x)*. The division is then multiplied with the average iodine concentration of the supplying blood vessels over all patients *ø(I_blood_)* in order to keep the units [mg/ml] and thus the measurability of the equation.


$$I_{L\_bn}(x)=\frac{I_{L\_abs}(x)}{I_{blood}(x)}*\emptyset (I_{blood})=\frac{I_{L\_abs}(x)}{I_{blood}(x)}*\frac{\sum_{i=1}^{n}I_{blood}(i)}{n}$$ (2)

In the equation *I_L_bn_(x)* denotes the blood normalized iodine concentration of a patient’s liver, *I_L_abs_(x)* denotes the absolute iodine concentration of the patient’s liver, *I_blood_(x)* denotes the absolute iodine concentration in the blood vessels of the patient and *øI_blood_* denotes the average absolute iodine concentration in the blood vessels over all patients of the study.

In order to evaluate different outcomes of a blood-normalized iodine concentration we set up three different calculation scenarios for *I_blood_(x)* and *øI_blood_*: the portal vein normalized iodine concentration *I_pv-n_(x)*, the aorta normalized iodine concentration *I_a-n_(x)* and the mixed blood normalized iodine concentration *I_mixed-n_(x)*. It is known that the hepatic portal vein delivers approximately 75% of the blood flow to the liver and the hepatic artery, arising from the aorta, supplies the resting 25% of the blood flow [19]. Therefore, we calculated the absolute iodine concentration of the mixed hepatic blood supply *I_mixed_blood_(x)* as:

$$I_{mixed\_blood}(x)=0.75*I_{portalvein}(x)+0.25*I_{aorta}(x)$$ (3)

Then, the three different blood-normalized iodine concentration were obtained as defined in Eq 2 by dividing the measured absolute iodine concentration of a patient’s liver *I_L_abs_(x)* parenchyma by the average absolute iodine concentration of the patient’s portal vein *I_portalvein_(x)*, aorta *I_aorta_(x)* or mixed blood *I_mixed_blood_(x)* respectively and then multiplying the division with the average iodine concentration of the corresponding vessels (i.e. aorta, portal vein or mixed) over all patients of the study.


$$I_{pv-n}(x)=\frac{I_{L_{abs}}(x)}{I_{portalvein}(x)}*{\emptyset (I}_{portalvein}),$$ (4)

$$I_{a-n}(x)=\frac{I_{L\_abs}(x)}{I_{aorta}(x)}*{\emptyset (I}_{aorta}),$$ (5)

$$\begin{array}{l} I_{mixed-n}(x)=\frac{I_{L\_abs}(x)}{I_{mixed\_blood}(x)}*{\emptyset (I}_{mixed\_blood})\\ \;=\frac{I_{L\_abs}(x)}{0.75*I_{portalvein}(x)+0.25*I_{aorta}(x)}*(0.75*\emptyset I_{portalvein}+0.25*\emptyset I_{aorta})\\ \;=\frac{I_{L\_abs}(x)}{0.75*I_{portalvein}(x)+0.25*I_{aorta}(x)}*\frac{\sum_{i=1}^{n}\left({0.75*I}_{portalvein}(i)+0.25*I_{aorta}(i)\right)}{n} \end{array}$$ (6)

### Statistical analysis

Statistical analysis was performed using statistics software (IBM SPSS Statistics, version 26.0, IBM). A non-parametric Mann-Whitney-U-test was used to compare the iodine concentration in liver parenchyma between male and female patients. The Pearson correlation analysis was used to evaluate the relation between iodine concentration and age and simple linear regression was performed to define the slope of corresponding the regression lines. For all tests used, a p value of < 0.05 was considered to be statistically significant. The range of iodine concentration in the healthy liver parenchyma was defined as the mean value +/- two standard deviations [μ-2σ; μ+2σ].

## Results

### Absolute iodine concentration values measured in liver parenchyma and blood supplying vessels

The absolute iodine concentration was measured in the segments 3, 4b and 7 of the liver parenchyma.

Table 1 shows the results of these measurements, indicating the average iodine concentration over all patients, and over the male and female study cohort for each of the liver ROIs. It may be appreciated that the iodine concentration in the three ROIs placed in different locations of the liver did show similar average iodine concentrations and standard deviations indicating that there is no evidence of regionally varying uptake of iodine in the parenchyma. Also, the table reveals that there are differences in the iodine concentration of male and female patients, with female patients showing a higher iodine uptake in the liver tissue than male patients.

**Table 1:** Average measured iodine concentration values in liver ROIs.

Iodine concentration [mg/ml]	All patients		Male patients		Female patients	
	Ø	σ	Ø	σ	Ø	σ
ROI S.3 (I_ROI s.3_)	2.072	0.548	1.876	0.493	2.357	0.504
ROI S.4b (I_ROI s.4b_)	2.098	0.539	1.921	0.486	2.355	0.514
ROI S.7 (I_ROI s.7_)	2.120	0.536	1.936	0.481	2.394	0.501

Mean values and standard deviations of the absolute iodine concentration in the ROIs of the three different liver segments for the entire study population as well as for male and female patients.

Table 2 shows the average iodine concentration measured in the supplying vessels (i.e., aorta and portal vein), also for the entire study cohort as well as for the male and female cohort correspondingly. The values show that the average iodine concentration was higher in the portal vein than in the aorta, which was to be expected due to the portal venous phase of the CT scan. In addition, the table shows that the average iodine concentration in the vessels, this is both aorta and portal vein, was also generally higher for female than for male patients.

**Table 2:** Average measured iodine concentration values in supplying vessels.

Iodine concentration [mg/ml]	All patients		Male patients		Female patients	
	Ø	σ	Ø	σ	Ø	σ
Aorta (I_aorta_)	4.382	1.029	3.942	0.799	5.031	1.000
Portal vein (I_portalvein_)	4.974	1.073	4.479	0.778	5.703	1.045

Mean values and standard deviations of the absolute iodine concentration in the aorta and portal vein for the entire study population as well as for male and female patients.

### Relation between patient gender and iodine concentration

The absolute iodine concentration of the liver tissue was significantly higher for female patients than for male patients (p < 0.001). The mean values and standard deviations for male and female patients are given in Table 3. All three calculated blood-normalized iodine concentrations did not show any significant difference between genders (p = 0.720 for *I_pv-n_*, p = 0.383 for *I_a-n_* and p = 0.584 for *I_mixed-n_*).

**Table 3:** Average measured iodine concentration values and standard deviations.

Iodine concentration [mg/ml]	All patients		Male patients		Female patients	
	Ø	σ	Ø	σ	Ø	σ
Absolute (I_abs_)	2.097	0.216	1.911	0.470	2.369	0.482
Portal vein normalized (I_pv-n_)	2.099	0.321	2.115	0.340	2.074	0.293
Aorta normalized (I_a-n_)	2.125	0.426	2.143	0.423	2.097	0.435
Mixed blood normalized (I_mixed-n_)	2.097	0.316	2.112	0.322	2.074	0.310

Mean values and standard deviations of absolute iodine concentration and blood-normalized iodine concentrations for the entire study population as well as for male and female patients.

**Fig 2:**
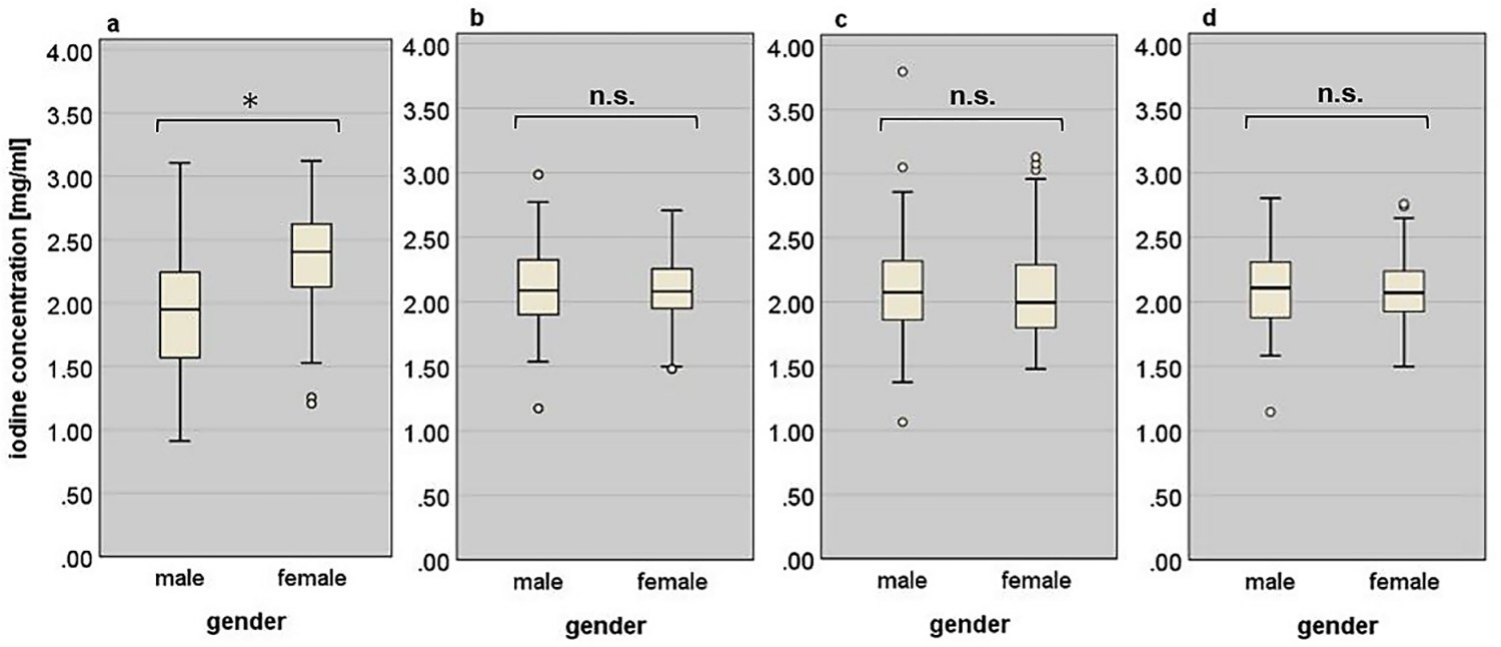
Quantitative iodine concentrations between genders. (a) The absolute iodine concentrations *I*_*L_abs*_*(x)* show a significant difference (p < 0.001) between genders. The portal vein normalized iodine concentration *I*_*pv-n*_*(x)* (b), the aorta normalized iodine concentration *I*_*a-n*_*(x)* (c) and the mixed blood normalized iodine concentration *I*_*mixed-n*_*(x)* (d) do not show any significant differences between male and female patients.

Fig 2 illustrates these results and shows the corresponding box plot diagrams comparing the absolute iodine concentrations as well as the different blood-normalized iodine concentrations between male and female patients.

### Relation between patient age and iodine concentration

The absolute iodine concentration of the liver tissue did not show a significant correlation with the patient age (r = -0.144, p = 0.080).

However, significant negative correlations were evidenced after normalizing the iodine concentration of the liver parenchyma with the iodine concentration of the blood. The blood-normalized iodine concentrations and the patients’ age showed negative correlations for all three blood-normalized concentration values (i.e., aorta-, portal vein- and mixed blood-normalized iodine concentration). The strongest correlations with the patients’ age were found for the portal vein normalized iodine concentration *I_pv-n_* and the mixed blood normalized iodine concentration I_mixed-n_ (r = -0.295, p < 0.01 and r = -0.295, p < 0.01 respectively). A smaller negative correlation with the patients’ age was also detected for the aorta normalized iodine concentration *I_a-n_* (r = -0.177, p = 0 < 0.05).

**Fig 3:**
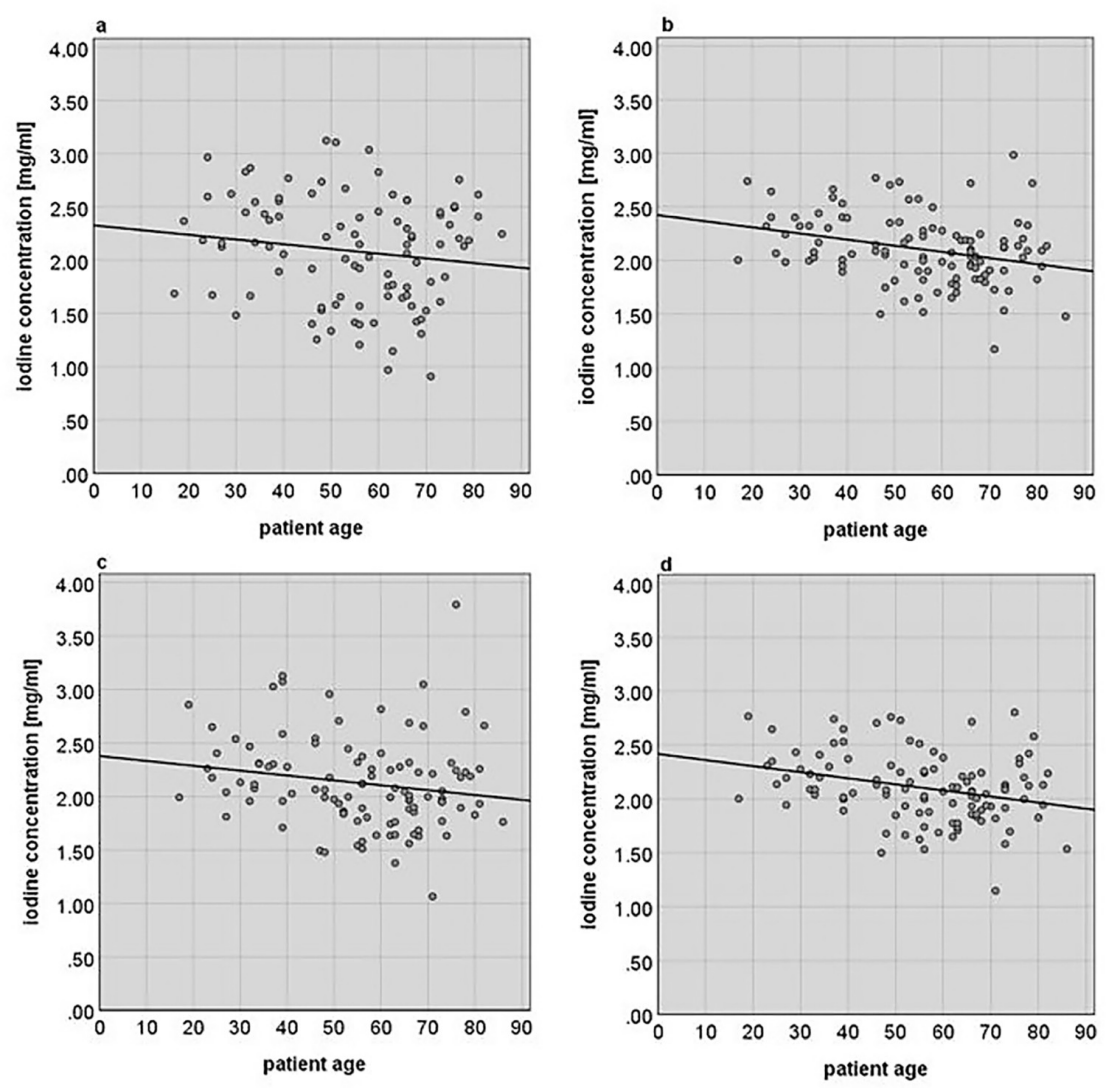
Scatterplot diagrams relating the measured and calculated iodine concentrations to the patients’ age. The measured absolute iodine concentration *I*_*L_abs*_*(x)* **(a)** does not show a significant correlation with the patients’ age (R^2^ = 0.021, r = -0.144, p = 0.080), while the different calculated blood-normalized iodine concentrations **(b)–(d)** do show a clear and significant negative correlation with the patients’ age. The portal vein normalized iodine concentration *I*_*pv-n*_*(x)* **(b)** shows a correlation of R^2^ = 0.087 (r = -0.295, p < 0.01). The aorta normalized iodine concentration *I*_*a-n*_*(x)* **(c)** shows a correlation of R^2^ = 0.031 (r = -0.177, p < 0.05). The mixed blood normalized iodine concentration *I*_*mixed -n*_*(x)* **(d)** shows a correlation of R2 = 0.087 (r = -0.295, p < 0.01).

Fig 3 shows the scatterplot diagrams of the absolute iodine concentrations and the different blood-normalized iodine concentrations and their variation with the patients’ age.

**Fig 4:**
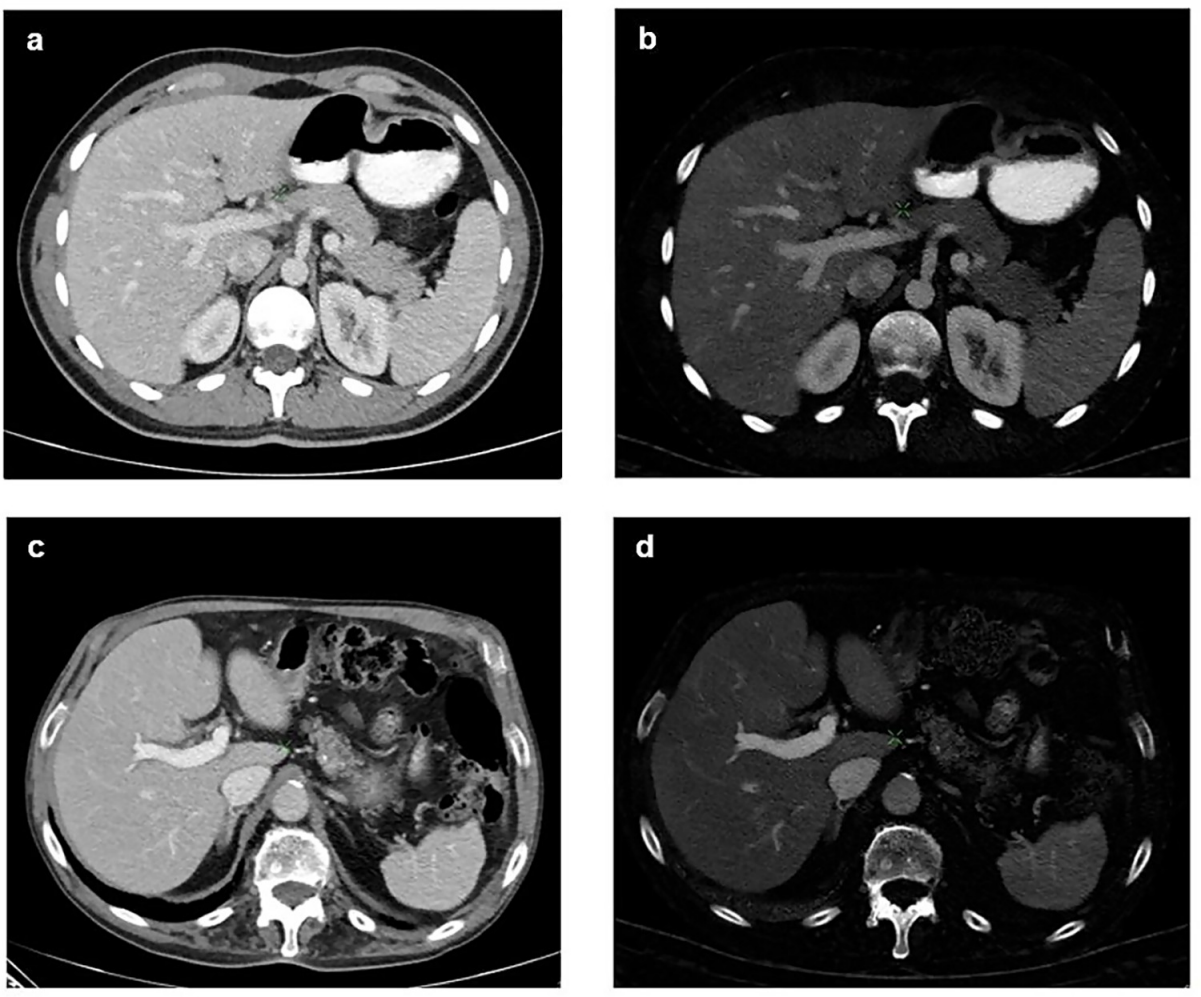
Comparison of iodine uptake of young and elderly patient. Fig **(a)** and **(b)** show the HU and iodine concentration DECT images of a young patient aged 20 years. Fig **(c)** and **(d)** show the HU and iodine concentration DECT images of an elderly patient aged 79 years. Although the iodine concentration in the portal vein of the young patient in fig **(b)** is lower than the iodine concentration in the portal vein of the elderly patient in fig **(d)**, the iodine concentration of the liver parenchyma is visibly higher in the liver parenchyma of the younger patient **(b)** than it is for the elderly patient **(d)**. The different uptake of contrast agent is clearly visible in the iodine images **(b)** and **(d)** while it is not that impressive in the HU images **(a)** and **(c)**.

Fig 4 gives an example of DECT liver scans of 2 patients, one of age 20 and one of age 79. Both scans were performed 70 seconds after contrast injection. The quantitatively assessed iodine concentration of the liver parenchyma is visibly higher for the younger patient. While the HU images show very similar density of the liver parenchyma of both patients, the difference of the iodine concentration is clearly visible in the iodine image. Please note there are differences in the iodine concentration in the portal vein and aorta between both patients, indicating different systemic perfusion parameters.

### Definition of standard values for absolute iodine concentration of the liver parenchyma

A significant difference in the absolute iodine concentration of the liver parenchyma of male and female patients was observed. Therefore, gender specific ranges should be established for the evaluation of absolute iodine concentrations. For female patients, with an average absolute iodine concentration of 2.369 mg/ml and a standard deviation of 0.482 mg/ml, the range was established between 1.405 mg/ml and 3.332 mg/ml. For male patients, with an average absolute iodine concentration of 1.911 mg/ml and a standard deviation of 0.470 mg/ml, the range was established between 0.970 mg/ml and 2.852 mg/ml.

Since no correlation between absolute iodine concentration and age was found, the patients’ age seems to have no measurable impact on the absolute iodine concentration value range.

### Definition of standard values for blood-normalized iodine concentration of the liver parenchyma

After normalizing the absolute iodine concentration of the liver parenchyma with the iodine concentration of the blood supplying vessels, the difference of the iodine concentration between genders was eliminated. No further significant difference was found for any of the three blood-normalized iodine concentrations between male and female patients. Therefore for both, female and male patients, the physiological range for the portal vein normalized iodine concentration has been defined between 1.457 mg/ml and 2.742 mg/ml (ø = 2.099 mg/ml, σ = 0.321 mg/ml), the physiological range for the aorta normalized iodine concentration has been defined between 1.272 mg/ml and 2.978 mg/ml (ø = 2.125 mg/ml, σ = 0.426 mg/ml) and the physiological range for the mixed blood normalized iodine concentration has been defined between 1.465 mg/ml and 2.729 mg/ml (ø = 2.097 mg/ml, σ = 0.316 mg/ml).

As to the correlation with the patients’ age, a negative correlation between iodine concentration and the age was revealed after normalizing the absolute iodine concentration of the liver parenchyma with the iodine concentration of the supplying blood.

Since the average age of the patients of our study population was 56 years, the above defined ranges are most suitable for patients of this age. For younger and older patients, the physiological blood normalized iodine concentration ranges should be increased or decreased accordingly to the slope of the simple linear regression line.

According to the slopes of the linear regression lines of our population’s scatter graphs, the range for the portal vein normalized iodine concentration should be increase by 0.0057 mg/ml for every year that a patient is younger than 56 years and reduced by 0.0057 mg/ml for every year a patient is older than 56 years. The corresponding variability of the aorta normalized iodine concentration and the mixed normalized iodine concentration range is of 0.0046 mg/ml and 0.0056 mg/ml per year respectively.

Table 4 shows an overview of the observed standard physiological iodine concentration ranges.

**Table 4:** Observed physiological iodine concentration ranges.

Iodine concentration [mg/ml]	Male patients	Female patients
Absolute (I_abs_)	[0.970–2.852]	[1.405–3.332]
Portal vein normalized (I_pv-n_)	[1.457–2.742] (± 0.0057 / year)	
Aorta normalized (I_a-n_)	[1.272–2.978] (± 0.0046 / year)	
Mixed blood normalized (I_mixed-n_)	[1.465–2.729] (± 0.0056 / year)	

Observed average physiological standard value ranges for absolute and blood-normalized iodine concentration of the healthy liver parenchyma for a patient aged 56 years and variability per year of age.

## Discussion

The direct and quantitative measurement of iodine concentrations with DECT offers more precise and reliable information about contrast enhancement than the common HU measurement values since it avoids the superposition of CT values of the contrast medium and the body tissue.

This study shows that the iodine concentration of the liver parenchyma after contrast medium uptake is influenced by age and by gender. Different absolute iodine concentrations were detected between male and female patients but disappeared when normalizing the iodine concentration values to the blood iodine concentration. The absolute iodine concentrations did not show a correlation with the patient’s age, but an existing correlation turned visible when normalizing the iodine concentration of the liver parenchyma with the iodine concentration of the supplying vessels.

In this study, we presented a first analysis of standard value ranges for quantitative iodine concentrations of the healthy liver parenchyma in portal venous phase MD iodine images in DECT after injection of iodine contrast agent.

Besides the absolute iodine concentrations in the liver parenchyma indicated by DECT image analysis, we calculated blood-normalized iodine concentrations, relating the absolute iodine concentration of the liver parenchyma to iodine concentration in the blood supplying vessels (portal vein, the aorta and mixed supply). The blood-normalized iodine concentrations reduce the side effects of patient specific physiological and anatomical singularities, such as hemodynamics, total blood volume, stroke volume, cardiac output, etc. [20, 21]. Therefore, we consider the normalized iodine values to have a higher pertinence than the absolute iodine values. Since the CT images were acquired in portal venous phase, it is consequential, that the mixed blood normalized iodine concentration and the portal venous normalized iodine concentration show better results than the aorta normalized iodine concentration. The lowest standard deviation was obtained for mixed blood normalized iodine concentration values.

The results of the measurements showed a significant difference of the absolute iodine concentration vales of the liver parenchyma between genders.

Though while the female patients showed a higher absolute iodine concentration of the liver parenchyma than male patients, they also showed a higher iodine concentration in the supplying vessels (i.e., aorta and portal vein), as shown in Table 2.

The gender specific differences in the absolute iodine concentration of the liver parenchyma disappeared by normalizing the absolute iodine concentration of the liver parenchyma with the iodine concentration of the supplying vessels. No significant difference remained between the iodine concentration of male and female patients after the normalization in any of the three blood-normalized measurements.

It may therefore be concluded that the higher absolute iodine concentration measured in the liver parenchyma of the female cohort in our study is directly caused by the higher iodine concentration in the supplying vessels and not by the nature of the liver parenchyma itself.

We assume that the difference of the absolute iodine concentrations between male and female patients in the patents’ blood and hence in the liver parenchyma originate mainly in the gender specific physiological characteristics of the circulatory system, such as hemodynamics, total blood volume, stroke volume and cardiac output [20, 21].

We therefore conclude that there is no physiological difference in the actual contrast medium uptake of the liver parenchyma between female and male patients but only a difference in the iodine supply to the liver parenchyma. This hypothesis is accounted for by the statistically equal blood normalized iodine concentration values for both genders. When measuring absolute iodine concentrations of the liver parenchyma, it is therefore reasonable to relate the measured values to the iodine concentrations of the supplying blood vessels.

The analysis of the relation between the iodine concentration of the liver parenchyma and the patients’ age showed no significant correlation for the absolute iodine concentration values. A significant negative correlation was however detected for the blood-normalized iodine concentrations and thus after eliminating a possible confounding bias caused by differences in the iodine concentration of the blood supply.

We therefore assume that the fact that no significant correlation could be demonstrated for the absolute iodine concentration values, is due to the prominent influence of the physiological singularities of each patient on the blood supply of the liver tissue (i.e., hemodynamics, total blood volume, stroke volume, cardiac output) and in consequence on the absolute iodine uptake of the liver tissue. These singularities seem to outweigh the rather small impact of the age, making it undetectable in the measurement of absolute iodine values. Since the impact of these singularities of the circulatory system and the corresponding confounding bias is reduced by relating the liver iodine concentration to the blood supply, the correlation shows in the blood-normalized iodine concentrations. The fact that the correlation is only measurable for blood normalized values shows that the correlation does not depend on the age-specific features of the circulation but on the age-specific properties of the liver tissue itself. This again proves the blood-normalized values to be more precise than the absolute iodine values. The correlation between age and blood-normalized iodine concentration of the healthy liver parenchyma should be considered when considering iodine concentration values for diagnostic matters.

Generally, whenever possible, blood normalized iodine concentration values should be used for diagnostic evaluation of the iodine uptake and iodine concentration of the liver parenchyma.

There are several limitations to this study. First, the variability observed with age and gender in the measured absolute and blood-normalized iodine concentrations was not confirmed by or correlated to any histologic findings from tissue sampling. Second, the age distribution of the scanned patients does not show a uniform distribution but is slightly skewed towards higher ages.

Our work is based on a monocentric study with images of one single DECT platform which limits the generalization of the results. Therefore, in the future, further studies with additional participating centers should be conducted to validate the reliability of the values presented in this work.

Also, the defined standard value ranges of our study, and especially those of the absolute iodine concentrations, are highly influenced by the used CT protocol and its various factors, such as type and amount of contrast agent, the method of contrast administration and the imaging delay. This may influence the comparability of the results of our study with values obtained by other studies using different parameters and protocols.

Furthermore, the accuracy of the quantitative iodine concentrations measured by DECT may be subject to variations caused by the technical differences between the dual energy platform used, this is to say dual layer CT, dual source CT or rapid kVP switching CT. In addition, the accuracy could be influenced by systemic variations caused by scanning protocols and varying patient size or weight.

The accuracy of iodine measurements with different DECT platforms has been addressed in the previous publications, and the potential influence could be determined to have only marginal influence in the system used here [6–8]. For example, Sellerer, et al. [9] have shown in a comparison phantom study, that all DECT systems measure iodine concentrations with a very high accuracy, especially in lower iodine concentration ranges of under 4 mg/ml, which is the range that applies to the iodine concentrations measured in the liver tissue in our study. Nevertheless, the different technologies do show slightly different mean quantification errors, although especially for higher iodine concentration ranges, for which reason future studies should analyze and compare the in vivo impact of different DECT systems on the measured iodine concentration of the liver.

Ehn, et al. [8] have analyzed the impact of body size and weight on the accuracy of iodine concentration measurements with dual layer CT in a phantom study and have shown that increasing body mass leads to only small variations in the measurement accuracy. Therefore, we do not expect a relevant impact of the varying patients’ weight on the iodine measurement accuracy.

Nevertheless, the physiological iodine uptake of the liver parenchyma could potentially vary with different patients’ weights. In our study we limited the study cohort to patients with a normal body weight comprehended between 55 and 75 kg in women and between 70 and 90 kg in men. Further studies should compare the iodine uptake of patients with higher and lower body weights and analyze the correlation of weight and iodine uptake of the liver parenchyma.

## Conclusion

Quantitative standard value ranges in portal venous phase material density iodine images in Dual Energy CT images can be determined for absolute and blood-normalized iodine concentrations in healthy liver parenchyma. The ranges for absolute iodine concentration vary with the patients’ gender mainly due to differences in the iodine concentrations of the blood supplying vessels. This gender specific differences can be eliminated by relating the measured absolute iodine concentration of the liver tissue to the iodine concentration of the blood supplying vessels, revealing no gender specific differences in iodine concentration of the liver parenchyma for the blood-normalized iodine concentrations.

The iodine uptake and resulting concentration of the liver parenchyma varies also with the patients’ age, although this effect is rather small and therefore only measurable after eliminating the impact of the varying iodine concentrations of the blood supplying vessels and therefore in the blood-normalized iodine concentrations but is not detectable when observing the absolute iodine concentrations measured in the liver parenchyma. The blood normalized iodine concentrations might therefore be a robust biomarker for assessing deviations due to various diseases (e.g., systemic liver diseases, oncology).

## Supporting information

S1 Table Raw measurement data set. The table shows the measurement HU and iodine values in the three ROIs, the aorta and portal vein along with the corresponding standard deviation of each measurement, as well as the relevant anonymous patient data i.e., age and gender.
(XLSX)
